# Supplementary figures and images for: Effects of afforestation with Pinus sylvestris var. mongolica plantations combined with enclosure management on soil microbial community
Source: PeerJ. 2020 Mar 25;8:e8857. doi: 10.7717/peerj.8857 (PMC7102505; doi:10.7717/peerj.8857)

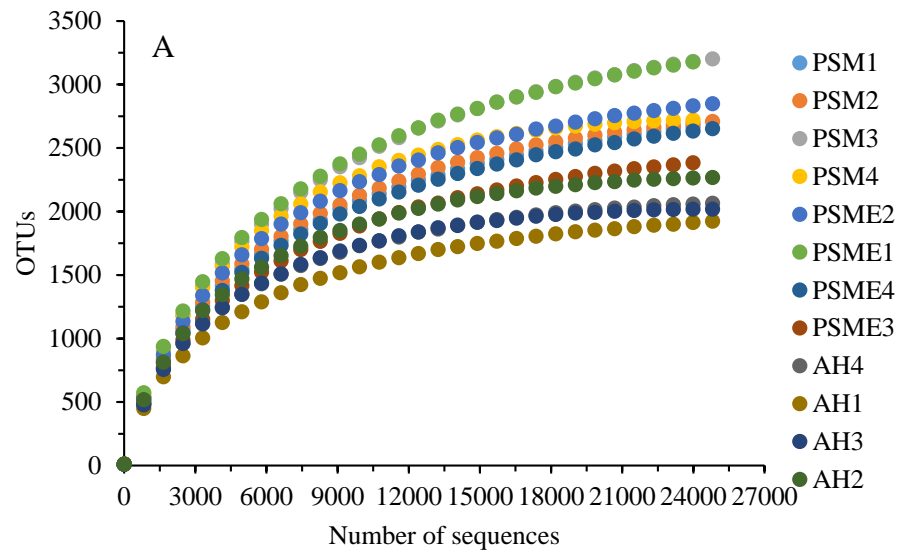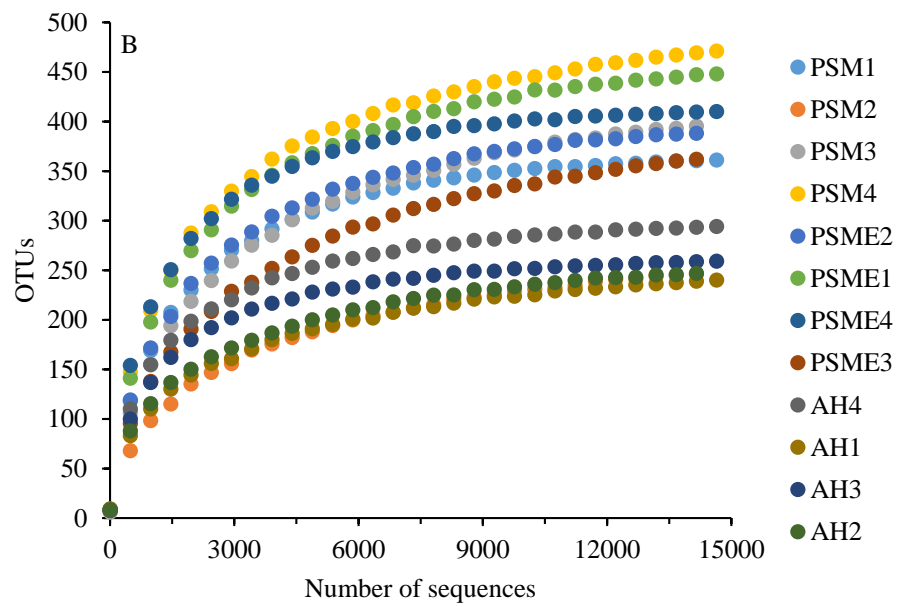

Supplement: Supplemental Information 1 — AH: Arachis hypogaea; PSM: Pinus sylvestris var. mongolica; PSME: Pinus sylvestris var. mongolica with enclosure. [file peerj-08-8857-s001.pdf]
